# RECOMMENDATIONS TO IMPROVE AGING PAIN INTERVENTION RESEARCH ON MINORITIES: A SCOPING REVIEW

**DOI:** 10.1093/geroni/igae098.3033

**Published:** 2024-12-31

**Authors:** Avani Shah, McKenzie Brown

**Affiliations:** University of Alabama, Tuscaloosa, Alabama, United States; University of Alabama, Tuscaloosa, Alabama, United States

## Abstract

Limited information is available for evidence supported psychological treatment options for pain in minority populations. The purposes of this scoping review were to determine if psychological treatments were 1) evaluated for minority populations; 2) if minorities were sufficiently included in clinical trials 3) if there were trials that were specific to minority populations. A search was conducted in PsycINFO, MEDLINE, and CINAHL databases for psychological interventions for pain in older adults over the age of 50. We also searched review articles. Search terms were chronic pain OR pain; older adults OR elderly OR geriatric OR aged OR human; pain management OR nonpharmacological pain management OR intervention OR psychotherapy; randomized control trial. Papers had to be in English, a randomized clinical trial, with pain as the primary outcome, and the treatment had to be a psychological intervention. We coded studies that had at least a 90% minority sample as a trial specific to a minority population or if one of the groups was minority. From 14,000 studies identified by the search, we reviewed 485 full texts. Of those,129 psychological interventions studies on older adults with chronic pain met inclusion and exclusion criteria. There were 6 African-American, 7 Asian,1 Turkish, and 2 Iranian samples. Studies represented 24 countries. However, 72 studies did not provide enough information about ethnicity or race. The lack of reporting of race and ethnicity took place across many disciplines and journal types. All journals should require reporting ethnic and racial demographics of participants to increase generalizability of findings.

